# Information Filtering via a Scaling-Based Function

**DOI:** 10.1371/journal.pone.0063531

**Published:** 2013-05-17

**Authors:** Tian Qiu, Zi-Ke Zhang, Guang Chen

**Affiliations:** 1 School of Information Engineering, Nanchang Hangkong University, Nanchang, P. R. China; 2 Institute of Information Economy, Hangzhou Normal University, Hangzhou, P. R. China; 3 Web Sciences Center, University of Electronic Science and Technology of China, Chengdu, P.R. China; 4 Beijing Computational Science Research Center, Beijing, P. R. China; Jacobs University Bremen, Germany

## Abstract

Finding a universal description of the algorithm optimization is one of the key challenges in personalized recommendation. In this article, for the first time, we introduce a scaling-based algorithm (SCL) independent of recommendation list length based on a hybrid algorithm of heat conduction and mass diffusion, by finding out the scaling function for the tunable parameter and object average degree. The optimal value of the tunable parameter can be abstracted from the scaling function, which is heterogeneous for the individual object. Experimental results obtained from three real datasets, *Netflix*, *MovieLens* and *RYM*, show that the SCL is highly accurate in recommendation. More importantly, compared with a number of excellent algorithms, including the mass diffusion method, the original hybrid method, and even an improved version of the hybrid method, the SCL algorithm remarkably promotes the personalized recommendation in three other aspects: solving the accuracy-diversity dilemma, presenting a high novelty, and solving the key challenge of cold start problem.

## Introduction

Favored by increasing information, people can enjoy an abundant life. However, people are also brought into a quandary decision of getting what they actually prefer. For example, how to select a satisfactory dress from various dress brands, or get an interesting book to read from the book sea. As a powerful tool, recommendation engine emerges to help people out of the overloaded information [1]. With the need of personalized recommendation, developing efficient recommendation methods has become one of the central scientific programs.

A great many algorithms have been proposed, and have led to a considerable progress, such as the collaborative filtering (CF) algorithms [2], [3] which can be further divided into memory-based [4]–[6] and model-based methods [7]–[10], content-based algorithms [11]–[14], and the relevant extensive studies [15]–[21]. Recently, favored by the fruitful achievements of complexity theory, complex-network-based recommendation algorithms have been proposed [21], [22], which directs a promising way for the personalized recommendation [23]–[35]. Meanwhile, concepts from traditional physical domain have been introduced into the algorithm design, e.g., the introduction of the thought of mass diffusion [24], [28] and heat conduction [23], [28], which greatly promotes recommendation accuracy and diversity, respectively.

Among these numerous physical-concept-based recommendation algorithms, a representative work is a hybrid algorithm of heat conduction and mass diffusion (HHP) [28]. Generally, improving the recommendation accuracy usually inhibits the recommendation diversity. However, the need of personalized recommendation resorts to a powerful engine that is not only accurate but also personalized. Whereas improving the recommendation accuracy, the HHP method simultaneously elevates the recommendation diversity, which therefore greatly contributes to solving the long-standing dilemma between the recommendation accuracy and diversity for the network-based recommender systems. Inspired by this work, extensive methods have been proposed in various disciplines, such as the integrated weighted tags [36] and the target-drug prediction [37]. A promising direction of improvement is to consider the heterogeneity of users or objects [38], which might lead to a more personalized recommendation matching individual tastes.

However, for a number of different algorithms, the algorithm performance is usually controlled by some ‘tunable parameter’. What challenges these algorithms in common is how to find out the optimal value of the tunable parameter. By far, most algorithms take a one-evaluator-based parameter selection, namely, choosing the optimal value of the tunable parameter according to the recommendation performance of one evaluator [28], [35], [39], [40]. For instance, one can take the value of the tunable parameter as its optimal value, with which parameter the system leads to its best recommendation accuracy. Nevertheless, without bias, different recommendation focuses might prefer different evaluator performance. Consequently, a challenging question emerges: which evaluator is the best one to be used as the reference of searching for the optimal value of the tunable parameter? Even though the recommendation accuracy is widely accepted to be the most important evaluator in personalized recommendation, the cold start problem or the recommendation diversity and novelty also raises a central interest [28], [41], . The cold start problem refers to how to recommend the new object or recommend the interesting object to new users due to the lack of activity records. The diversity and novelty also significantly mark the vitality of a system. Explicitly, one can hardly find out the same optimal value of the tunable parameter according to different recommendation focal purposes. Moreover, even when evaluating the recommendation accuracy, different indicators might reach different optimal values of the tunable parameter. For example, the ranking score [24] and the precision [43] are both indicators which are used to evaluate the recommendation accuracy. However, the optimal value of the tunable parameter obtained from the ranking score and the precision are usually not consistent for the same method.

Motivated by the explicit dilemma to choose a proper reference of the algorithm optimization, in the present paper, for the first time, we introduce a scaling-based algorithm (SCL) independent of the recommendation list length, based on the hybrid method of heat conduction and mass diffusion (HHP). By testing our algorithm on three real datasets, *Netflix*, *MovieLens* and *RYM*, we here report two results:

1. A single curve independent of the recommendation list length is obtained by rescaling the tunable parameter and the object average degree, and we describe it by a scaling function. The optimal value of the tunable parameter can be abstracted from the scaling function, which is heterogeneous for the individual object.
2. The present algorithm shows a high accuracy in recommendation. More importantly, it greatly improves the personalized recommendation in three other challenging aspects: solving the accuracy-diversity dilemma, presenting a high novelty, and solving the cold start problem.

The remainder of this paper is organized as follows. In the next section, we detail the bipartite network and the investigated recommendation algorithms. Some popular indicators to evaluate the recommendation algorithm performance are introduced in the section of metrics, and followed by the description of the datasets in the data section. Then, we compare the results of the present algorithm with a highly accurate mass diffusion algorithm, the original both highly accurate and diverse hybrid method, and even an improved version of the hybrid method which well resolves the cold start problem in the section of results and discussion. Finally comes to the conclusion.

## Materials and Methods

A recommendation system can be described by a bipartite network composed of a user set and an object set. The user set includes 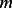 users 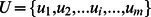, and the object set includes 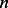 objects 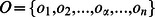. If an object 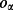 is collected by a user 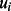, then add a link between them. The adjacent matrix which links the users and the objects is 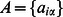. If the object 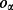 is collected by the user 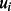, then 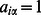, otherwise, 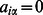.

In the following algorithms, a so-called “resource” is introduced to objects. At first, objects are assigned an initial resource **f**, with 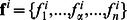 for a particular user 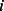. If an object is collected by the user 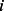, its initial resource is assigned to be 1, otherwise, to be 0. That is to say, for the user 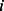, the initial resource 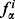 of the object 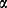 equates the value of the adjacent matrix element 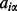, i.e., 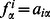. After a resource reallocation process via a transformation matrix **W**, objects obtain a final resource 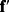 formulated by 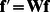. For each user, rank his/her uncollected objects in the decreasing order of the final resource, and then recommend the top 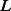 objects to the user. The formula of the transformation matrix 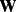, i.e., how to redistribute the resources, therefore plays a key role in the recommendation process.

### PBS and HTS Methods

**Figure 1:**
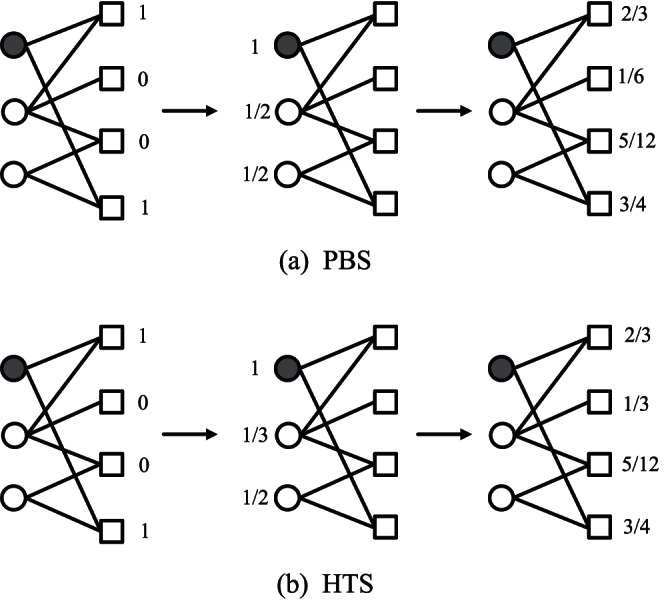
An illustration of the resource reallocation process. (a) for the PBS method, and (b) for the HTS method.

The mass-diffusion based algorithm, referring to the probability spreading (PBS) process based algorithm, is reported as a highly accurate method [24]. An example is illustrated in figure 1 (a) to show the process of the resource reallocation. Initially, the four objects are assigned a resource. At first, each object distributes the resource to its neighboring users with an equal probability. For example, for the particular user indicated by the solid circle with two neighboring objects, i.e., the first and the fourth object. The first object transits 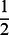 resource to the user, and the fourth object also transits 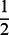 resource to the user. Therefore, the user can get the total resource of 1 from his/her neighboring objects. Then the user again redistributes the total resource of 1 to his/her neighboring objects with the equal probability, i.e., the first and the fourth objects both get 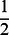 resource from the user. By summing up all the resources from their neighboring users, the objects then obtain their final level of resources. The resource transformation matrix of the PBS is formulated as,
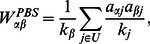
(1)where 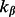 is the degree of object 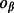, and 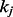 is the degree of user 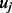 (Degree is denoted as the number of links owned by the user or the object). We assume an object to be popular if the object has a high degree, otherwise, the object to be cold. In the last step of the PBS, due to objects receiving resources from all their neighboring users, it greatly upgrades the resources of objects with high degrees. Henceforth, the PBS assigns more priority to the popular objects, leading to a good recommendation accuracy, yet a relatively low diversity.

By incorporating heat-conduction analogous process, the heat conduction (HTS) method is proposed, with an illustration of how resources are reallocated shown in figure 1 (b). Firstly, the user gets the average resource from all his/her neighboring objects. For example, for the particular user indicated by the solid circle, he/she receives 1 resource from the first object and 1 resource from the fourth object. Taking an average over the two objects, the user therefore gets the total resource of 1. Then the object again gets the average resource from all its neighboring users. The transformation matrix then reads,
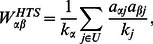
(2)where 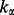 is the degree of object 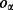. In the last step of the HTS, due to the resources of objects divided by their degree, the rank of objects with high degrees is greatly depreciated. Therefore, the HTS assigns more priority to the cold objects, leading to a good performance in recommendation diversity, but at the cost of the recommendation accuracy.

### Hybrid Method and an Improved Version

To achieve a high accuracy and diversity of recommendation, a hybrid method (HHP) is proposed [28], by elegantly combining the heat conduction and the mass-diffusion method as,
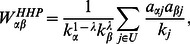
(3)where 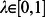. When tuning the parameter 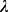 to a suitable value, the HHP method shows an apparent advantage in both the recommendation accuracy and the diversity.

Based on the HHP method, an improved object-oriented hybrid method (OHHP) is proposed [38], focusing on resolving the cold-start problem. In the OHHP, an object-degree-dependent tunable parameter is introduced, with its resource transformation matrix to be,
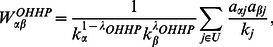
(4)where 
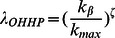
, 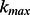 is the maximal degree of all the object degrees, and 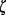 is a tunable parameter. The OHHP actually optimizes the probability spreading factor in the transformation matrix of equation (3) according to the individual object degree level, therefore it greatly enhances the recommendation accuracy of cold objects, whereas keeping a high recommendation accuracy of the overall objects.

### Scaling-based Method

**Figure 2:**
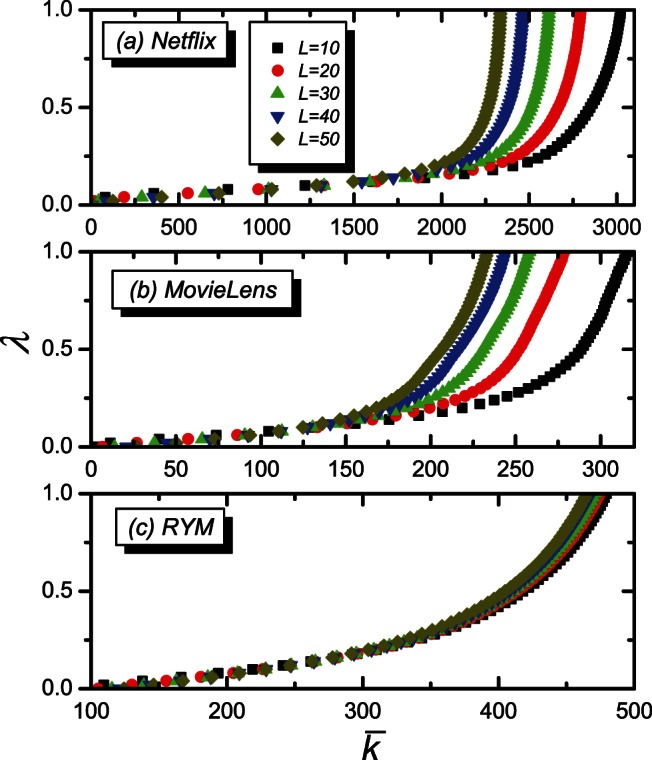
The tunable parameter 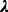 on the object average degree 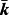. The black, red, green, blue and dark yellow lines are for the recommendation list lengths of 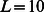, 20, 30, 40 and 50, respectively.

The common question in most algorithms is how to find out the optimal value of the tunable parameter. For example, the optimal value obtained by utilizing the ranking score as the reference is usually different from that obtained by utilizing the diversity as the reference. Moreover, diversity performance varies with the recommendation list length. We show the tunable parameter 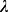 on the object average degree 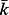 for different recommendation list length 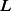 in the HHP algorithm in figure 2, where 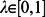. For three real datasets, the *Netflix*, *MovieLens*, and *RYM* (Details of the datasets will be introduced in the Data section), 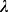 on 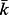 exhibits different behavior for different recommendation list length. It indicates that, for different recommendation list length, one can obtain different value of the tunable parameter for the same object average degree. If the scaling behavior independent of the recommendation list length can be found, the tunable parameter on the object average degree for different recommendation list length can be then described in a universal way.

In order to obtain an 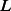-independent scaling function, we analytically investigate the recommendation result for the HHP algorithm. On average, the probability that a user 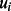 collects an object 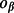 is directly proportional to 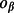
’s degree, 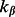, that is to say, 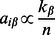, where 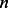 is the number of objects. Hypothesize that the probability of 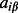 is independent of other links. For the particular user 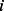, the resource 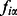 of the object 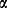 can be calculated according to the transformation matrix, which reads,
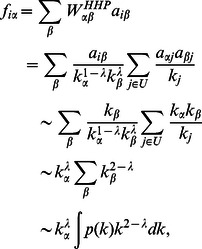
(5)where 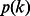 is the probability distribution function of the object degrees. As suggested in Ref. [38], 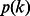 obeys a power-law distribution from the empirical study, i.e., 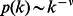. Then, one can calculate 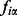 as,
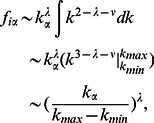
(6)where 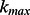 and 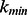 are respectively the maximum and the minimum of the object degrees.

**Figure 3:**
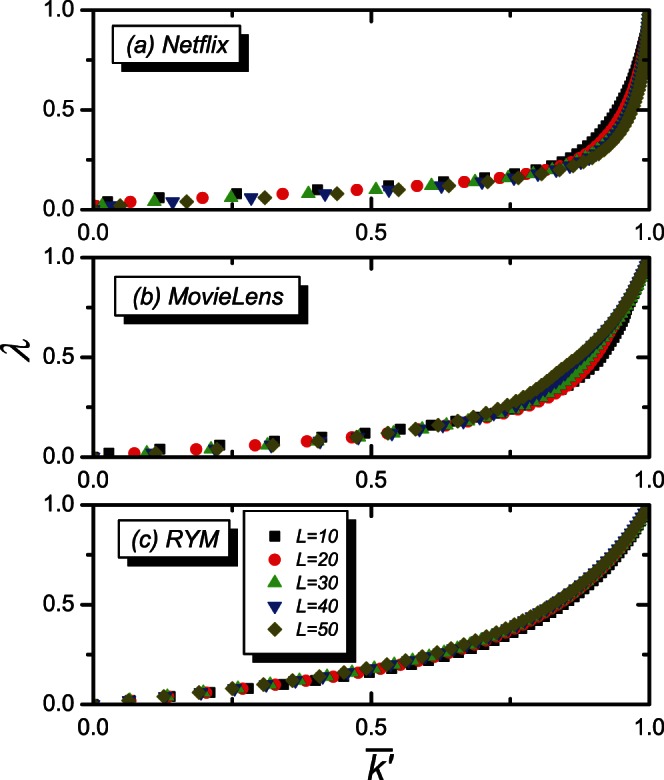
The rescaled tunable parameter 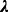 vs. the rescaled object average degree 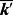. The black, red, green, blue and dark yellow lines are for the recommendation list lengths of 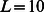, 20, 30, 40 and 50, respectively.

Inspired by the above theoretical analysis, we propose the Scaling-based (SCL) algorithm, making use of the formula in equation (6) to collapse the data into a single curve characterized by the scaling form,
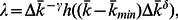
(7)where 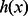 is a universal function, 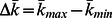, with 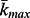 and 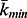 to be the maximum and minimum of the object average degree 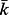 for the overall range of 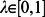. We rescale the axes 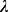 and 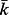 according to the transformation 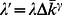 and 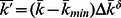, and obtain 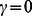 and 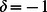 to make all the curves roughly collapsed to a single curve. Therefore, 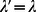 and 
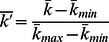
. As shown in figure 3, the major part of the curves is well collapsed. However, due to the fluctuations of empirical data, a small part of the curves is only approximately collapsed. The procedure to obtain the optimal value of the tunable parameter in the SCL is as follows:

1. Make the polynomial fit 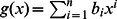 for the single curve, so that one can obtain a set of fitting coefficients 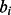, where 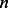 is the number of polynomial fitting order. Here we take 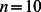 to obtain the coefficient set 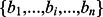.
2. Having the coefficients 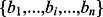, compute the optimal value of the tunable parameter 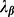
for a particular object 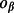 according to the formula 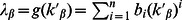, where 
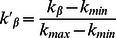
, with 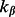 being the degree of the examined object 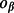, 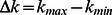, and 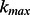
(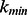) being the maximal (minimal) degree of all the objects.
3. Having the optimal value of the tunable parameter 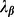 for a particular object 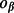, calculate its resource transformation matrix as

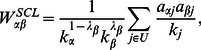
(8)Henceforth, the optimal value of the tunable parameter in the SCL is no longer accessed according to any specific evaluator, but abstracted from the scaling function acquired from the single curve.

### Metrics

Recommendation accuracy is with no doubt one of the most important indicators to evaluate the performance of an algorithm. As an adjunct to accuracy, recommendation diversity and novelty are addressed to be important evaluators to quantify the personalized recommendation. In our study, we take the ranking score, precision and recall to quantify the recommendation accuracy, the object average degree to quantify the novelty, the inter-diversity and inner-diversity to quantify the recommendation diversity. Moreover, to specifically investigate the recommendation accuracy of cold objects, we further study an object-dependent ranking score, an object-dependent precision, and an object-dependent recall.

#### 1. Ranking score (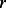) [24]

The ranking score 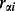 for the object 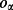 to the user 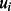 is defined as,
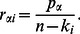
(9)where 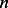 is the number of all objects, 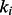 is the degree of the user 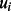, and 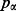 is the position of the recommended object 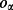 located in all the uncollected objects of the user 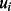. Generally speaking, users collect the objects which they prefer. Namely, for a user 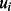, if the deleted link with an object 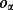 is in a higher rank of 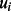
’s all deleted links, the algorithm is more accurate. The average ranking score 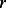 is then defined as the average of 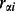 over all the deleted links. The smaller the 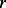, the more accurate the algorithm.

To focus on the recommendation accuracy of cold objects, we define an object-degree dependent ranking score 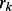 as the average ranking score over objects with the same value of degrees [39].

#### 2. Precision (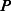) [43]

The recommendation precision 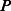 is defined as
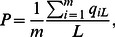
(10)where 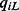 is the number of the user *u_i_*’s deleted links contained in the top 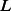 recommended object list. The larger the 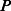, the higher accuracy the algorithm.

Similarly, to better understand the recommendation accuracy of the cold objects, we define an object-degree dependent precision by,
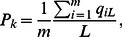
(11)where 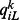 is the number of the user *u_i_*’s deleted links for objects with degree 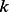 in the top 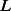 recommended object list.

#### 3. Recall (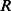) [43]

The recall 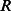 is defined as
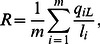
(12)where 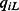 is the number of user *u_i_*’s deleted links contained in the top 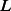 recommended object list, 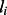 is the number of user *u_i_*’s deleted links in the test set.

The object-degree dependent recall is analogously defined as,
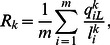
(13)where 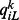 is the number of user *u_i_*’s deleted links for objects with degree 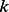 in the top 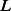 recommended object list, and 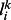 is the number of user *u_i_*’s deleted links for objects with degree 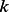 in the test set.

#### 4. Novelty (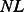)

The average degree of objects in the recommendation list is widely used to identify the novelty of a recommender system, which is defined by,
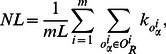
(14)where 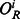 is the object set of user 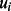
’s recommendation list. If 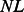 is small, it indicates that, on average, the degree of the recommended objects is small, i.e., more cold objects are recommended, which is therefore more novel to users; otherwise, if the recommended objects are on average with high degree, i.e., the popular objects, it is less novel to users.

#### 5. Inter diversity (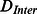)


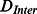
 quantifies the difference between different users recommendation list by
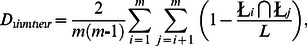
(15)where 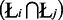 is the number of common recommended objects for user 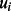 and 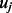 in the top 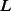 recommendation list. Generally, the greater the 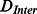, the more personalized the recommendation for different users, and vice versa.

#### 6. Inner diversity (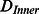)


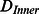
 calculates the difference within a specific user recommendation list by
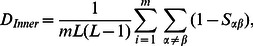
(16)where 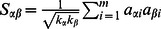 is the cosine similarity between objects 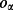 and 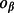 in a single user’s top 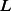 recommended object list. Generally, the greater the 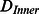, the higher diversification of the recommendation list for a specific user, and vice versa.

### Data

We test the algorithm performance on three datasets, the *Netfilx*, *MovieLens* and *RYM*. The *Netflix* and *MovieLens* are movie rating systems with a five-level rating and the *RYM* is a music rating system with a ten-level rating. The *Netflix* dataset is obtained by randomly selecting from the huge dataset of the *Netflix* Prize, and the *MovieLens* is downloaded from the web site of GroupLens Research (http://grouplens.org), and the *RYM* dataset is downloaded from the music rating web site RateYourMusic.com. Due to the different level of ratings, we perform a coarse-graining mapping to a unary form for all the three datasets. If the rating is no less than three for the *Netflix* and *MovieLens*, and six for the *RYM*, we argue that the object is collected by a user. The *Netflix* contains 9999 users, 5870 objects and 815917 links, and the *MovieLens* contains 943 users, 1682 objects and 100000 links, and the *RYM* contains 10159 users, 5250 objects and 559634 links. The sparsity of the datasets, defined as the number of links proportional to the total number of the user-object links, is 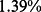, 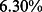 and 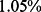 for the *Netflix*, the *MovieLens* and the *RYM*, respectively.

We divide a dataset into two subsets of the training set and the test set. We randomly delete 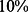 links as the test set, and remain the rest 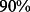 links as the training set. We utilize the training set to make predictions for users, and the test set to test the algorithm performance.

## Results and Discussion

To provide a solid investigation of the performance of the SCL algorithm, we compare the performance of the SCL with three typical and excellent algorithms, the PBS, the HHP, and the OHHP. The PBS is highly accurate, and the HHP well resolves the great challenge of accuracy-diversity dilemma, and the OHHP further outperforms the HHP in resolving the cold start problem. A summary of the performance of the PBS, the HHP, the OHHP and the SCL is presented in table 1, with the results being the average over six runs.

**Table 1:** The performance of the PBS, HHP, OHHP and SCL methods.

		*r*	*r_k≤_*_10_	*P*	*P_k≤_*_10_	*R*	*R_k≤_*_10_	*NL*	*D_Inter_*	*D_Inner_*
*NET*	PBS	0.051	0.484	0.054	0.0000	0.420	0.0003	2336.0	0.637	0.423
	HHP	0.045	0.417	0.062	0.0006	0.470	0.0176	1843.7	0.720	0.672
	OHHP	0.044	0.350	0.058	0.0009	0.437	0.0255	2048.3	0.691	0.575
	SCL	0.046	**0.357**	0.060	**0.0012**	0.426	**0.0340**	**1497.5**	**0.792**	**0.768**
*MOV*	PBS	0.105	0.562	0.074	0.0000	0.477	0.0000	233.5	0.645	0.616
	HHP	0.083	0.408	0.085	0.0011	0.527	0.0441	157.2	0.717	0.839
	OHHP	0.083	0.364	0.084	0.0015	0.528	0.0527	170.6	0.707	0.818
	SCL	0.087	**0.326**	0.080	**0.0028**	0.469	**0.0928**	**128.2**	**0.762**	**0.881**
*RYM*	PBS	0.069	0.480	0.042	0.0002	0.497	0.0080	465.7	0.829	0.874
	HHP	0.048	0.250	0.050	0.0024	0.557	0.0924	329.7	0.850	0.940
	OHHP	0.050	0.189	0.047	0.0048	0.542	0.1578	374.8	0.849	0.919
	SCL	0.050	**0.168**	0.048	**0.0055**	0.539	**0.1835**	**317.9**	**0.862**	**0.941**

The overall ranking score 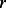, the object-degree dependent ranking score 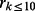, the overall precision 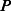, the object-degree dependent precision 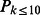, the overall recall 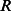, the object-degree dependent recall 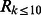, the novelty 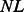, the inter-diversity 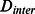 and the inner-diversity 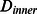 of the PBS, HHP, OHHP and SCL methods are shown for the *Netflix*(*NET*), the *MovieLens*(*MOV*) and the *RYM*, with 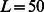.

To detect how much the SCL outperforms the other three algorithms, we define an improvement percentage 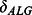 by,
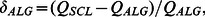
(17)where the subhead 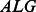 refers to the investigated algorithm, and the 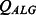 is the value of the indicator, i.e., the value of 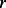, 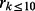, 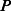, 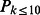, 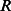, 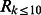, 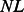, 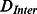 and 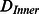. The improvement percentage 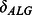 of the SCL against the PBS, the HHP and the OHHP is summarized in table 2.

**Table 2:** The improvement percentage of the SCL against the PBS, HHP and OHHP methods.

		*r*	*r_k≤_*_10_	*P*	*P_k≤_*_10_	*R*	*R_k≤_*_10_	*NL*	*D_Inter_*	*D_Inner_*
*NET*	*δ_PBS_*	**9.8%**	**26.2%**	**11.1%**		**1.4%**	**11233.3%**	**35.9%**	**24.3%**	**81.6%**
	*δ_HHP_*	−2.2%	**14.4%**	−3.2%	**100.0%**	−9.4%	**93.2%**	**18.8%**	**10.0%**	**14.3%**
	*δ_OHHP_*	−4.5%	−2.0%	**3.4%**	**33.3%**	−2.5%	**33.3%**	**26.9%**	**14.6%**	**33.6%**
*MOV*	*δ_PBS_*	**17.1%**	**42.0%**	**8.1%**		−1.7%		**45.1%**	**18.1%**	**43.0%**
	*δ_HHP_*	−4.8%	**20.1%**	−5.9%	**154.5%**	−11.0%	**110.4%**	**18.4%**	**6.3%**	**5.0%**
	*δ_OHHP_*	−4.8%	**10.4%**	−4.8%	**86.7%**	−11.2%	**76.1%**	**24.9%**	**7.8%**	**7.7%**
*RYM*	*δ_PBS_*	**27.5%**	**65.0%**	**14.3%**	**2650.0%**	**8.5%**	**2193.8%**	**31.7%**	**4.0%**	**7.7%**
	*δ_HHP_*	−4.2%	**32.8%**	−4.0%	**129.2%**	−3.2%	**98.6%**	**3.6%**	**1.4%**	**0.1%**
	*δ_OHHP_*	0.0%	**11.1%**	**2.1%**	**14.6%**	−0.6%	**16.3%**	**15.2%**	**1.5%**	**2.4%**

The improvement percentage of the SCL against the PBS, HHP and OHHP in the overall ranking score 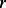, the object-degree dependent ranking score 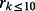, the overall precision 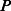, the object-degree dependent precision 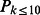, the overall recall 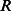, the object-degree dependent recall 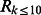, the novelty 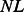, the inter-diversity 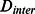 and the inner-diversity 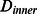 are shown for the *Netflix*(*NET*), the *MovieLens*(*MOV*) and the *RYM*, with 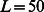. To guide the eyes, if the indicator of the SCL outperforms other methods, we show the improvement percentage as a positive value, otherwise, as a negative value. The blank in the form indicates an infinite value owing to the zero value of the PBS’s precision and recall.

From table 1 and table 2, for all the three datasets, the SCL shows a great advantage in recommendation accuracy of the low-degree objects, as well as novelty and diversity, while simultaneously keeping a high recommendation accuracy.

For the recommendation accuracy, we focus on the overall recommendation accuracy and the recommendation accuracy of the cold objects. Compared with the highly accurate PBS method, the SCL outperforms the PBS for almost all the metrics. Taking the *Netflix* as an example, the SCL outperforms the PBS as much as 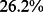 and 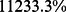 for the recommendation accuracy of the low-degree objects 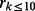 and 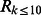; 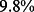 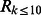 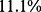 and 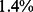 for the overall recommendation accuracy 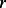, 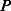 and 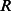; 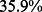 for the novelty 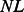; 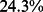 and 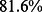 for the inter-diversity 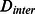 and the inner-diversity 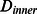. Due to the zero value of the 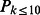 of the PBS, the improvement of the SCL against the PBS leads to an infinite value for the 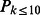. Similar outstanding performance of the SCL against the PBS is also observed for the *MovieLens* and the *RYM*. It indicates the SCL is highly accurate.

The HHP is excellent in both the accuracy and the diversity at the optimal value of the tunable parameter. Compared with the HHP at the optimal value of the tunable parameter evaluated by the ranking score, the SCL presents a very little lower overall recommendation accuracy, but a much greater advantage in the recommendation accuracy of the cold objects. Moreover, the SCL outperforms the HHP in the novelty 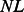, as well as both the inter-diversity 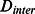 and the inner-diversity 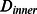 for all the three datasets. Taking the *Netflix* as an example, the HHP is 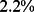 more advantageous than the SCL in the overall ranking score. However, the ranking score for the cold objects 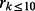 of the SCL is 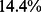 more advantageous than the HHP, and the improvement of the SCL against the HHP is as high as 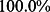 and 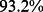 for the precision 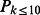 and recall 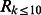 for the cold objects. It also suggests that the SCL is outstanding in the cold start problem, while keeping a high recommendation accuracy. To be significant, the improvement of the SCL against the HHP in the novelty 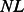, the inter-diversity 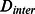 and the inner-diversity 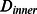 reaches 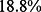, 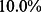 and 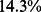, respectively.

The OHHP method has been reported to be more advantageous in the cold start problem than the HHP. Compared with the OHHP at the optimal value of the tunable parameter defined by the ranking score, the SCL method further improves the recommendation accuracy of the cold objects. Also, the SCL outperforms the OHHP in the novelty, the inter-diversity and the inner-diversity for all the three datasets.

The cold start problem is a long-standing challenge in traditional recommendation system, since it is difficult for users to be aware of the cold objects due to the lack of sufficient accessorial information [42]. Basically, the cold start problem can be divided into two categories [44]: i) *cold user* start [45] and ii.) *cold object* start [46]. The former focuses on recommending objects for new users, while the latter tends to design algorithms to push new objects, which is exactly what we are trying to solve in this paper. Most of researches in this area try to generate recommendation by using additional information, such as trust relationship [47], social network structure [48], tags [21], [30], [31], [41], [49], [50], etc [51]. However, it increases the system complexity. In addition, for most systems, the cold objects occupy a big proportion. In the *Netflix*, *Movielens* and *RYM*, the cold objects whose degrees are no more than 10 are as much as 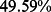, 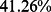, and 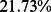. Developing effective information filtering techniques is essentially required to solve the cold start problem. Without any additional information, the SCL greatly improves the recommendation accuracy of the cold objects.

**Figure 4:**
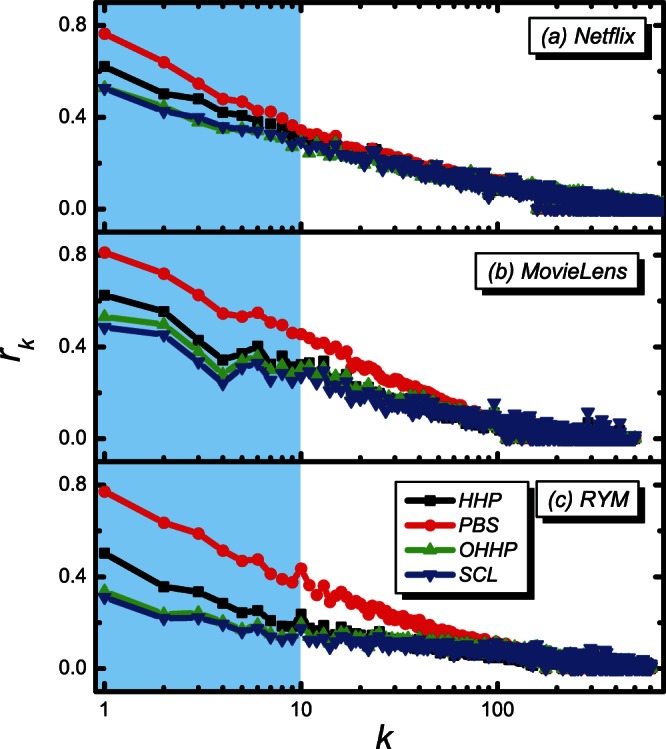
The object-degree dependent ranking score 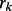 vs. the object degree. The black, red, green and blue lines are for the HHP, PBS, OHHP and SCL methods, respectively.

To further understand the cold start efficiency of the four algorithms, we investigate the object-degree-dependent ranking score 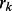 vs. the object degree 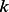. As shown in figure 4, it is observed that, the 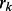 of the low-degree objects of the SCL is much smaller than that of the PBS and the HHP for all the three datasets, and even a little smaller than that of the OHHP for the *MovieLens* and the *RYM*, while keeping a close value for the popular objects with high degrees. It suggests that the SCL significantly elevates the recommendation accuracy for cold objects.

**Figure 5:**
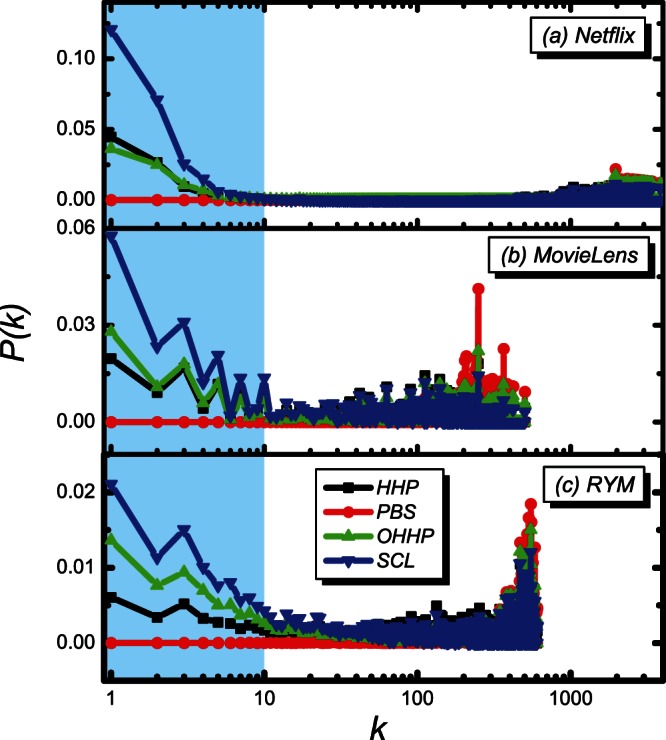
The degree distribution 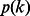 of the objects in the top 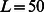 recommendation list. The black, red, green and blue lines are for the HHP, PBS, OHHP and SCL methods, respectively.

We then study the degree distribution 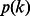 of the objects in the top 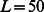 recommendation list in figure 5. It is observed that the 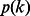 of the cold objects of the SCL is much greater than the PBS, the HHP and the OHHP, which indicates that the SCL indeed contributes greatly to the recommendation efficiency of the cold objects.

Besides the cold start problem, diversity and novelty are also significant to mark the vitality of personalized recommendation. Recommendation accuracy and diversity has been addressed to a dilemma pair, as well as accuracy-novelty. Typical examples are the PBS and HTS algorithms, where the PBS is more accurate but less diverse and novel, whereas the HTS is more diverse and novel but less accurate.

**Figure 6:**
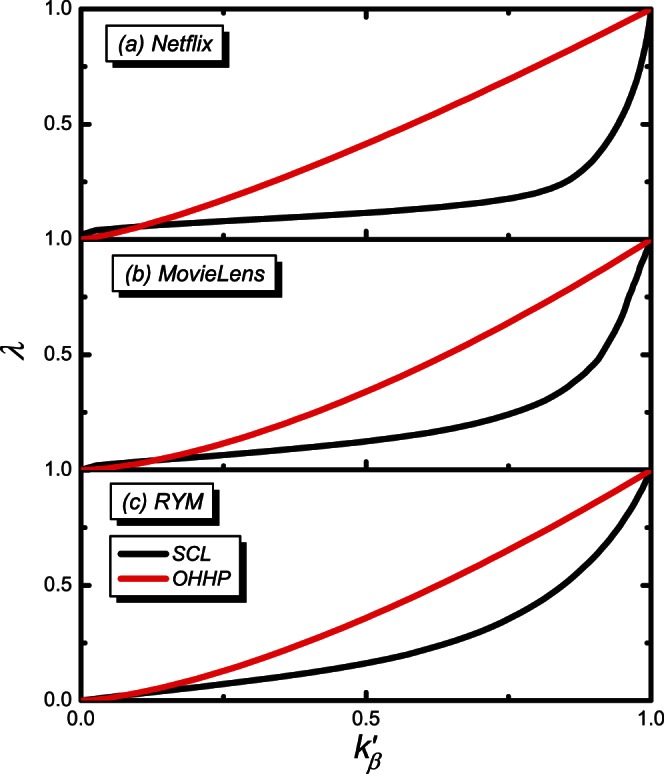
The tunable parameter 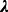 on the object degree 
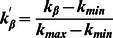
. The black and red lines are for the SCL and OHHP methods, respectively.

Intuitively, the improvement of recommendation accuracy of the cold objects would meanwhile upgrade the recommendation novelty and diversity. However, by comparing the OHHP with the original HHP, we find that the novelty, the inter-diversity and the inner-diversity of the HHP outperform those of the OHHP for all the three datasets, though the OHHP greatly improves the recommendation accuracy of the cold objects. To better understand the observed phenomena, we show the optimal value of the tunable parameter on the object average degree of the OHHP and the SCL in figure 6, where the curve of the SCL is obtained from the empirical study. It is observed that the curve obtained from the SCL is more heterogeneous than that obtained from the OHHP, which can partially explain why the OHHP method unilaterally improves the recommendation accuracy of the cold objects, but not simultaneously enhances the recommendation novelty and diversity. Compared with the OHHP, the SCL not only further improves the recommendation accuracy of the cold objects, but also elevates the recommendation novelty and diversity.

**Figure 7:**
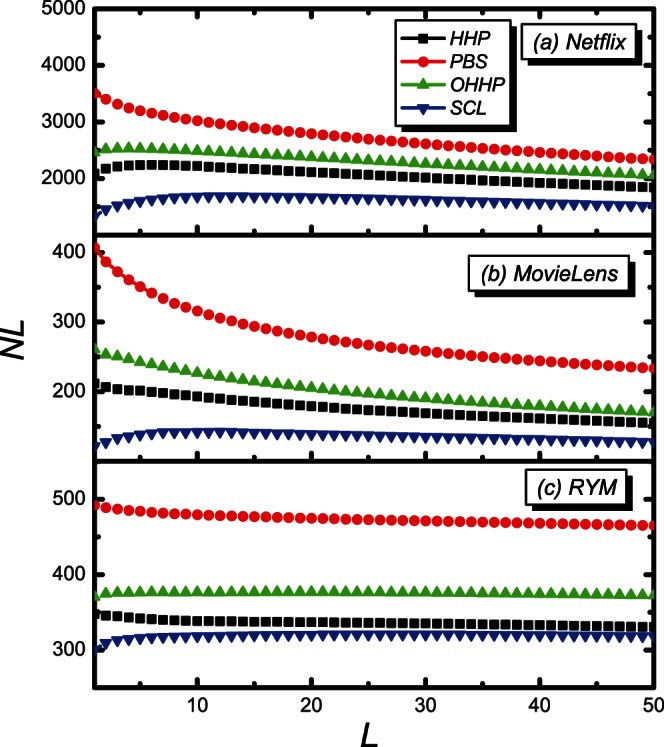
The novelty 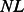 on the recommendation list length 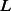. The black, red, green and blue lines are for the HHP, PBS, OHHP and SCL methods, respectively.

To manifest how the novelty evolves with the recommendation list length, we then study the novelty 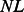 on the recommendation list length 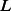. As shown in figure 7, for all the three datasets, the 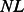 of the SCL is much smaller than that of the PBS, the HHP and the OHHP for all the investigated range of the recommendation list length. Also, the novelty of the SCL keeps quite stable with the recommendation list length for all the three datasets. It supports that the novelty of the SCL is quite advantageous.

**Figure 8:**
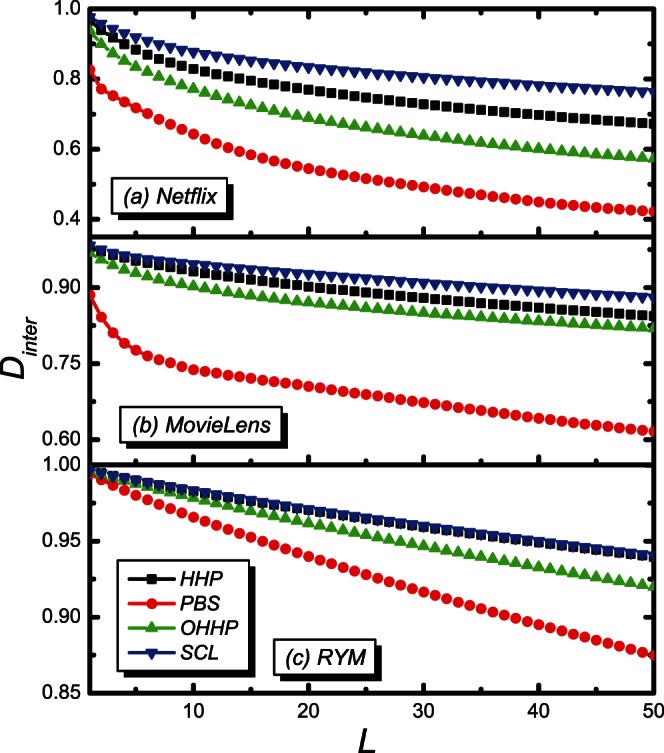
The inter-diversity 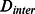 on the recommendation list length 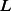. The black, red, green and blue lines are for the HHP, PBS, OHHP and SCL methods, respectively.

Further investigation of the inter-diversity 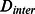 on the recommendation list length 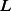 suggests that, for all the four methods, the inter-diversity decreases with the recommendation list length 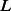, as shown in figure 8. It is reasonable since the difference between different users’ recommendation list would decrease with the augment of the recommendation list length 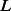. Compared with the PBS, the HHP and the OHHP, the SCL exhibits a much higher value. Moreover, the inter-diversity of the SCL shows a slower decay for the overall range of the recommendation list length 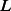 for the *Netflix* and the *MovieLens*. For the *RYM*, the inter-diversity 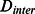 of the SCL is also higher than that of the PBS and the OHHP, and similar to the HHP with the recommendation list length evolving. It also indicates that the recommendation diversity of the SCL is advantageous.

**Figure 9:**
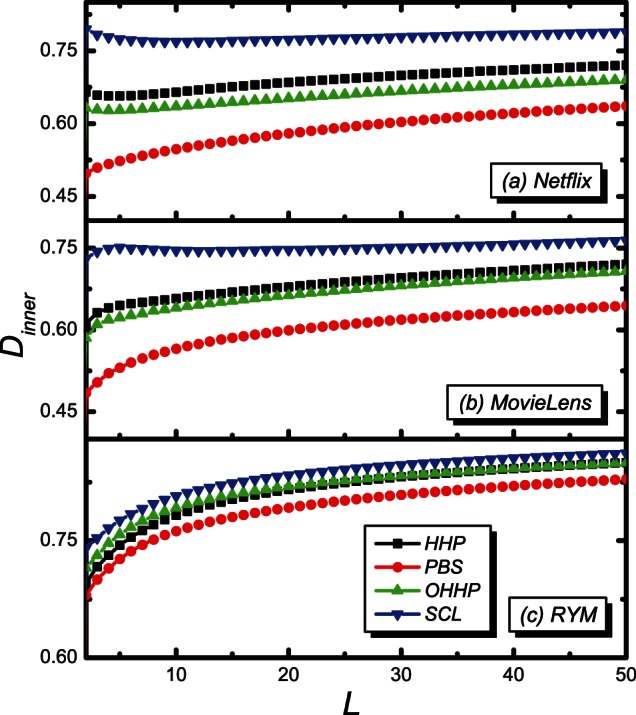
The inner-diversity 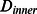 on the recommendation list length 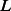. The black, red, green and blue lines are for the HHP, PBS, OHHP and SCL methods, respectively.

Similar advantage of the SCL is also found for the inner-diversity 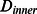, as shown in figure 9. It is observed that the 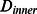 increases with 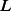 for all the four algorithms for the *Netflix*, the *MovieLens*, and the *RYM*, and the 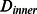 of the SCL is higher than the other three methods.

Taken together, while not searching for the optimal value of the tunable parameter according to any particular evaluator, but abstracting it from the scaling function, the SCL remarkably outperforms the PBS, the HHP, and the OHHP in the recommendation accuracy of cold objects, as well as the recommendation novelty and diversity, and simultaneously keeps a high overall recommendation accuracy.

### Conclusion

In conclusion, we have proposed a scaling-based (SCL) recommendation algorithm, in which the optimal value of the tunable parameter can be abstracted from the scaling function independent of the recommendation list length via a rescaled procedure. Based on three real datasets, *Netflix*, *MovieLens* and *RYM*, the optimal value of the tunable parameter is observed to be heterogeneous for the individual object in the SCL algorithm. Experimental results show that, the SCL algorithm not only shows a high accuracy, but also significantly promotes the performance in three other important aspects of personalized recommendation: improving the novelty, solving the long-standing cold start problem, as well as the accuracy-diversity dilemma.

The dilemma existing most in common in a number of algorithms is how to find out the proper value of the tunable parameter for different recommendation focuses, e.g., the accuracy, the diversity, or the cold start problem. It is with no doubt that recommendation accuracy is one of the most important evaluators of the algorithm performance. However, even using the recommendation accuracy as the reference to search for the optimal value of the tunable parameter, the optimal value might also be different for using different accuracy evaluators. By finding out a scaling function independent of the recommendation list length based on empirical data, we resolve the explicit dilemma of the optimal value selection of the tunable parameter for the complex contradiction among different recommendation focuses.
